# Effect of acupuncture on women with poor ovarian response: a study protocol for a multicenter randomized controlled trial

**DOI:** 10.1186/s13063-020-04690-8

**Published:** 2020-09-10

**Authors:** Huanfang Xu, Chensi Zheng, Liyun He, Tongsheng Su, Huidan Wang, Yu Li, Cui Zhao, Cuilian Zhang, Yang Bai, Guoqing Tong, Li Chen, Fang Zhao, Huisheng Yang, Mingzhao Hao, Yaqian Yin, Li Yang, Yigong Fang, Baoyan Liu

**Affiliations:** 1 Institute of Acupuncture and Moxibustion, China Academy of Chinese Medical Sciences, Beijing, China; 2 Institute of Basic Research in Clinical Medicine, China Academy of Chinese Medical Sciences, Beijing, China; 3 Shanxi Provincial Hospital of Chinese Medicine, Taiyuan, Shanxi China; 4 Shandong University Reproductive Hospital, Jinan, Shandong China; 5 Sun Yat-sen Memorial Hospital of the Sun Yat-sen University, Guangzhou, Guangdong China; 6 Lanzhou University First Hospital, Lanzhou, Gansu China; 7 Henan Provincial People’s Hospital, Zhengzhou, Henan China; 8 Huazhong University of Science and Technology Reproductive Medicine Center of Tongji Medical College, Wuhan, Hubei China; 9 Shanghai Shuguang Hospital, Shanghai, China; 10 East Region Military Command General Hospital, Nanjing, Jiangsu China; 11 Luoyang Women and Children Health Care Center, Luoyang, Henan China

**Keywords:** Acupuncture, Poor ovarian response, Number of retrieved oocytes, In vitro fertilization-embryo transfer, Randomized controlled trial

## Abstract

**Background:**

Poor ovarian response (POR), a manifestation of low ovarian reserve and ovarian aging, leads to a significant reduction in the pregnancy rate after in vitro fertilization-embryo transfer. Acupuncture has increasingly been used to improve the ovarian reserve. The purpose of this study will be to evaluate the effect of acupuncture on increasing the number of retrieved oocytes after controlled ovarian hyperstimulation in women with POR.

**Methods:**

This will be a multicenter randomized controlled trial. A total of 140 women with POR will be randomly assigned to receive acupuncture or nontreatment for 12 weeks before controlled ovarian hyperstimulation. The primary outcome will be the number of retrieved oocytes. The secondary outcomes will be antral follicle counts, serum levels of anti-Müllerian hormone, basal serum levels of follicle stimulating hormone, luteinizing hormone and estradiol levels, scores from the self-rating anxiety scale, fertilization rates, cleavage rates, available embryo rates, and high-quality embryo rates. The safety of acupuncture will also be assessed.

**Discussion:**

The results of this trial will help to determine the effectiveness of acupuncture in the treatment of POR. This may provide a new treatment option for patients with POR and their physicians.

**Trial registration:**

AMCTR-IPR-18000198. Registered on 10 August 2018.

## Background

Poor ovarian response (POR) indicates a reduction in follicular response to the controlled ovarian hyperstimulation (COH) during in vitro fertilization (IVF) cycles, and it results in a reduced number of retrieved oocytes [1]. It is believed that approximately 9–24% of women who undergo IVF show POR during COH [2]. Women with POR usually experience less follicular development in COH cycles, low blood estrogen levels, high doses of gonadotrophin, high cycle cancelation rates, decreased numbers of retrieved follicles, and low rates of clinical pregnancy [3]. Therefore, POR often leads to an unsatisfactory outcome in assisted reproductive technology (ART), which seriously affects physical and mental health as well as quality of life.

An individualized COH protocol designed according to the assessment of ovarian reserve is the key to success of POR treatment in the first and multiple cycles. Various protocols of ovarian stimulation, including gonadotrophin-releasing hormone (GnRH) agonist protocols [4], GnRH antagonist protocols [5], and some nontraditional protocols (microstimulation, natural cycles, luteal ovulation, etc.), have been proposed for optimizing ART outcomes; however, challenges remain for women with poor responses to COH. Many pretreatment modalities (including growth hormone, estrogen, dehydroepiandrosterone, and oral contraceptives) have been used prior to ovulation induction to increase the success rate for women undergoing ART [6–8], and yet evidence for the effectiveness of these pretreatments are limited [9].

Acupuncture, an important part of traditional Chinese medicine, has gained worldwide popularity for improving reproductive outcomes in women undergoing ART. Although high-quality research is still needed, several lines of evidence have shown that acupuncture is effective at increasing the number of retrieved oocytes [10], improving clinical pregnancy rate and live birth rate [11, 12], and relieving anxiety [13] in women who undergo in vitro fertilization-embryo transfer (IVF-ET). Low ovarian reserve often indicates a poor ovarian response in COH. Although acupuncture has not been studied for POR, its effectiveness in improving ovarian reserve for women with diminished ovarian reserve (DOR) or premature ovarian insufficiency (POI) has been reported. The results of our previous studies showed that acupuncture significantly lowered serum levels of follicle-stimulating hormone (FSH) and luteinizing hormone (LH), raised serum levels of anti-Müllerian hormone (AMH) and estradiol (E2), and increased antral follicle count (AFC) in patients with DOR [14, 15] or POI [16]. Acupuncture was found to regulate the menstrual cycle and to improve perimenopausal symptoms [14, 15].

Given its effect on the improvement of ovarian reserve and ART outcomes, acupuncture is expected to be a new choice for the treatment of POR. Furthermore, POR patients treated by transcutaneous electrical acupoint stimulation (TEAS), a noninvasive acupuncture-like therapy, showed significant improvement on basal serum sex hormones, AMH, the number of retrieved oocytes, and fertilization rate [17, 18], which indirectly indicated the effect of acupuncture for POR. Therefore, we aim to conduct a multicenter, randomized controlled trial (RCT) to evaluate the effectiveness of acupuncture on women with POR.

## Methods

### Study design

This will be an exploratory multicenter randomized controlled trial comparing acupuncture with nontreatment. A total of 140 women with POR will be recruited from the following 9 hospitals in China: Shanxi Provincial Hospital of Chinese Medicine, Shandong University Reproductive Hospital, Sun Yat-sen Memorial Hospital of the Sun Yat-sen University, Lanzhou University First Hospital, People’s Hospital of Henan Province, Huazhong University of Science and Technology Reproductive Medicine Center of Tongji Medical College, Shanghai Shuguang Hospital, Nanjing General Hospital of Nanjing Military Command, and Luoyang Women and Children Health Care Center. This protocol is in accordance with the principles of the Declaration of Helsinki. It was approved by the ethics committee of each participating hospital. Written informed consent will be obtained from patients prior to enrolment.

**Fig. 1:**
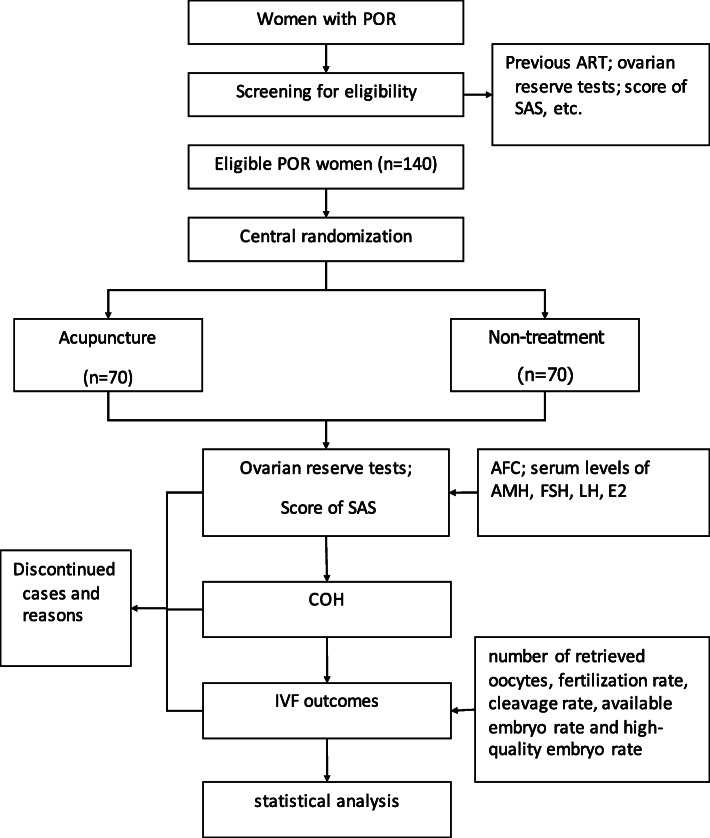
Trial flow chart

Eligible patients will be randomly assigned to receive acupuncture or nontreatment for 12 weeks, which will be followed by an IVF cycle. Outcomes will be assessed at baseline, after acupuncture or nontreatment intervention, and after the IVF cycle. The flowchart and study design schedule are presented in Fig. 1 and Table 1, respectively. This protocol has been registered at Acupuncture-Moxibustion Clinical Trial Registry (AMCTR-IPR-18000198).

**Table 1:** Study design schedule

Period	Screening	Baseline	Intervention	COH
Week (***W***)		***W***0	***W***12	***W***14
Informed consent	**×**			
Eligibility	**×**			
Demography and medical history	**×**			
AFC		**×**	**×**	
Serum level of AMH		**×**	**×**	
Basal serum levels of FSH, LH, and E2		**×**	**×**	
Score of SAS		**×**	**×**	
Number of retrieved oocytes				**×**
Number of fertilized oocytes				**×**
Number of cleavage				**×**
Number of available embryos				**×**
Number of high-quality embryos				**×**
AEs		**×**	**×**	**×**
Compliance		**×**	**×**	**×**

*AFC* antral follicle count, *AMH* anti-Müllerian hormone, *FSH* follicle-stimulating hormone, *LH* luteinizing hormone, *E2* estradiol, *SAS* self-rating anxiety scale, *AEs* adverse events

### Participants

We plan to recruit 140 women with POR from participating hospitals via poster, networking, or WeChat. The posters will be placed on bulletin boards or other assigned places in the hospitals. In each participating hospital, at least one staff member (usually a postgraduate or doctor) will specialize in patient recruitment. Their contact information and clinic for screening visit will be detailed described in the recruitment advertisement.

Participants will be included if they meet the following criteria: (1) diagnosis with POR according to the Bologna criteria published in 2011 by the European Society of Human Reproduction and Embryology [1]; (2) age < 40 years; (3) previous POR (≤ 3 oocytes retrieved in a conventional stimulation protocol); (4) an abnormal ovarian reserve test (i.e., AFC < 5–7 follicles or AMH < 0.5–1.1 ng/ml) or two episodes of POR after maximal stimulation; (5) suitability for GnRH antagonist protocol judged by physicians of reproductive medicine; and (6) voluntary participation and signing of the informed consent.

Participants will be excluded if they meet any of the following criteria: (1) history of ovarian surgery, radiotherapy or chemotherapy; (2) repeated miscarriages (including biochemical pregnancy) of more than 2 sessions; (3) repeated implantation failure (experienced at least 3 embryo transfer cycles, or failure to achieve clinical pregnancy after cumulatively transferring 10 good quality embryos); (4) uterine malformations (unicornate uterus, bicornuate uterus, uterus didelphys, or untreated uterus septus) or other untreated diseases that can damage the endometrial cavity such as adenomyosis, submucous myoma, intrauterine adhesions, and scarred uterus; (5) abnormal chromosomes in the patient or her partner (except chromosomal polymorphism); (6) untreated hydrosalpinx; (7) contraindications for pregnancy or ART; (8) untreated diseases that can negatively affect pregnancy such as high blood pressure, symptomatic heart disease, diabetes, liver or kidney disease, severe anemia, venous thrombosis, cerebral vascular diseases, and cancer; and (9) treatment with acupuncture or moxibustion treatment for POR in the previous 3 months.

### Randomization and masking

Block randomization stratified by center will be applied in this trial. Eligible participants will be randomly assigned to acupuncture or nontreatment groups in a 1:1 ratio. The scheme of randomization including any planned restriction (e.g., blocking) will be designed and archived by a statistician who will not be involved in the statistical work of this trial. The allocation sequence will be computer-generated. If a participant is eligible for the trial, the appointed researcher (acupuncturist or reproductive physician) in each center will apply for the random number and group assignment via a telephone- and internet-based central randomization system of the Clinical Evaluation Center of the China Academy of Chinese Medical Sciences. A strict view permission will be set for the central randomization system, which allows no one except the top system administrator to view its randomization scheme. In this study, outcome assessors and data analysts will be blinded to group allocation.

### Interventions

#### Acupuncture protocol

Patients in the acupuncture group will receive a 12-week acupuncture treatment regimen before COH. Acupuncture will be performed by registered acupuncturists with over 2 years of experience. Hwato brand disposable acupuncture needles (size 0.25 × 25 mm, size 0.25 × 40 mm, and size 0.30 × 75 mm) and SDZ-III electroacupuncture apparatuses (Suzhou Medical Appliance Factory, Suzhou, China) will be used.

The acupuncture protocol designed for this trial is based on our clinical practice for improving ovarian function for women with DOR [14]. It consists of two groups of acupoints: Group 1 (in supine position): GV20 (Baihui), GV24 (Shenting), GB13 (Benshen), CV12 (Zhongwan), ST25 (Tianshu), CV4 (Guanyuan), KI12 (Dahe), EX-CA1 (Zigong), SP6 (Sanyinjiao), KI3 (Taixi), LR3 (Taichong); and group 2 (in prone position): BL23 (Shenshu) and BL33 (Zhongliao). These two groups of acupoints will be used alternatively with group 1 as the initial treatment. All acupoints will be located according to the World Health Organization Standardized Acupuncture Points Location [19]. A needle depth of 10 to 20 mm will be applied for GV20, GV24, and GB13 with transverse insertion and for LR3 with oblique insertion to the direction of KI1 (Yongquan). BL33 will be needled with an oblique downward trajectory of approximately 60 to 70 mm into the third posterior sacral foramina. The remaining acupoints will be perpendicularly needled for approximately 30 to 40 mm. After insertion, small, equal manipulations of lifting, thrusting, or twirling will be performed on all needles (for GV20, GV24 and GB13, twirling only) to reach deqi, a composite of sensations (including soreness, numbness, distention, and heaviness) recognized as an essential component in achieving the acupuncture effect. In acupuncture with acupoints of group 2, paired electrodes from the electroacupuncture apparatus will be attached transversely to the needle handles at bilateral BL23 and BL33 with a dilatational wave and a current intensity that patients can tolerate (preferably with the skin around the acupoints shivering mildly without pain). Each acupuncture session will last for 20 min. Participants will receive 3 sessions of acupuncture per week (ideally every other day) for 12 consecutive weeks for a total of 36 sessions.

#### Nontreatment control

Participants in the control group will receive nontreatment for 12 weeks before COH.

#### COH regimen

Participant will start COH with a GnRH antagonist protocol on day 2 or 3 of the menstrual cycle after acupuncture or nontreatment period. GnRH antagonists are commonly used to prevent the premature LH surge that often occurs in poor responders [20, 21]. This induces pituitary suppression within a few hours without a flare-up effect, and the suppression is released immediately after their discontinuation [22]. Compared with GnRH agonist, GnRH antagonist protocol shows a shorter treatment time, a lower dosage of Gn, and a similar clinical pregnancy rate [5]. In this trial, individualized GnRH antagonist protocol will be administered by physicians of reproductive medicine in each center. Given that participants may become suitable for COH before they complete the 12-week acupuncture or nontreatment, an early termination of acupuncture or nontreatment aiming to seize the opportunity to initiate a COH cycle will be allowed on the condition that they complete at least the 4-week acupuncture treatment (12 sessions of acupuncture) or nontreatment period.

### Permitted and prohibited concomitant treatments

Participants will be discouraged from taking drugs that may interfere with the assessment of acupuncture effect, including Chinese herbs, Chinese patent medicine, sexual hormones, and contraceptives. If treatment not recommended in this trial has already been performed, relevant information should be recorded in patient’s case report form.

### Outcome measures

The primary outcome is the number of retrieved oocytes. The secondary outcomes include the assessment of ovarian reserve (AFC and serum levels of AMH, FSH, LH, and E2), other IVF outcomes (fertilization rate, cleavage rate, available embryo rate, and high-quality embryo rate), and the score of the self-rating anxiety scale (SAS). Ovarian reserve will be assessed at baseline and after acupuncture or nontreatment period. AFC (via transvaginal ultrasound) and serum levels of FSH, LH, and E2 are required to be measured on days 2 to 5 of the menstrual cycle. AMH can be measured on any day of the menstrual cycle. SAS is a self-rating scale for measuring the presence and severity of anxiety [23]. A validated Chinese version of SAS will be used [24]. It consists of 20 items rated on a 4-point Likert scale. When using the scale, participants will be asked to rate each item from 1 to 4 points according to how it applies to them within the past week. The standard score is the sum of the integer part of 1.25 times the raw score (the sum of the 20 items ranging from 20 to 80). The presence of anxiety symptoms is defined as an SAS standard score of greater than 50. A higher score indicates a more serious case of anxiety. SAS will be assessed at baseline and after acupuncture or nontreatment period.

### Assessment of safety

Adverse events (AEs) will be obtained by researchers by inquiring of the participants at each treatment or follow-up visit or by voluntarily reporting by the participants. Information of AEs will be appropriately documented throughout the trial. Acupuncture-related AEs include intolerable needling pain, bleeding after needle withdrawal, hematoma, and local infection. AEs related to COH include pain, organ injury, colporrhagia, and infection caused by egg retrieval, ovarian hyperstimulation syndrome, thrombosis, and allergic reaction.

### Data management and quality control

A pretrial training will be done for all participating staff on the trial protocol, acupuncture manipulation, usage of the central randomization, and data management systems, etc. Double data entry will be applied in this trial to ensure the accuracy of data entry. A three-level quality control system (including self-inspection of each center, monitoring by project management group, and auditing by a third party) will be applied to ensure the implementation, recording, and reporting of clinical trials conform to the trial protocol, standard operation specifications, and relevant laws and regulations. Self-inspection will be done at least once every month by the quality controller of each center. On-site or remote monitoring will be done for all centers once every 3 months by the monitors appointed by the principle investigator. The auditing will be done by Clinical Evaluation Center of the China Academy of Chinese Medical Sciences at the beginning, middle, and end of the trial. An ethics committee will review conduct especially on safety, rights, and well-being of the participants at the middle and the end of the trial. Regular reminders via WeChat will be used to improve participant compliance. For patients who discontinue or deviate from the protocol, causes and relevant outcome data will be recorded in a case report form as much as possible.

### Statistical methods

#### Sample size calculation

There are no available clinical studies on the effect of acupuncture for POR. Based on results of a TEAS study on POR [18], we assumed the mean (SD) number of retrieved oocytes would be 4.88 (1.84) in the acupuncture group and 3.95 (1.66) in the control group. A sample size of 57 participants per group would be needed to provide 80% power and a two-sided significance level of 5%. Allowing for a 20% dropout rate, 140 participants will be recruited with 70 participants per group.

#### Statistical analysis

Statistical analysis will be performed by a statistician blinded to group assignments using SAS version 9.4 (SAS Institute Inc). All analyses will be based on the intention-to-treat principle. Missing data will be imputed using the multiple imputation method. Continuous data will be presented as the mean and standard deviation, or the median and interquartile range; categorical data will be presented as the number and percentage. Comparisons between groups will be analyzed using an independent *t*-test or Wilcoxon rank-sum test for continuous variables, and a chi-square test or Fisher exact test for categorical variables. All statistical tests will be two-sided, and *p* < 0.05 will be considered statistically significant.

## Discussion

This trial aims to determine whether acupuncture can improve follicular response to the COH during IVF cycles for women with POR. Several studies have shown that acupuncture improved ovarian reserve and reproductive outcomes in women undergoing IVF. However, the study population of these trials generally did not focus on women with POR. To date, there is only one study (a protocol published in 2018) [25] that aimed to assess the effect of acupuncture for POR. The present trial will be the second study using acupuncture treating POR. Compared with the published POR research [25], this trial will use the same control (nontreatment) and primary outcome (the number of retrieved oocytes), but a different acupuncture protocol and more secondary outcomes to better assess the acupuncture effect for POR. In this trial, nontreatment rather than placebo will be used as control due to the following reasons: placebo acupuncture is difficult to implement [26], and women are usually suggested to rest for 2–3 months between the two IVF cycles in clinical practice. It is worth mentioning that the acupuncture protocol of this trial is a mature protocol that has been used in our clinical practice for more than 8 years, and it has been shown to be effective in improving ovarian function [14, 15].

There are some limitations in this trial. We will only include women with POR aged under 40 who are suitable for GnRH antagonist protocol, which may weaken the representative nature of the sample. Based on clinical practice, the acupuncture protocol comprising two series of acupoints seems a little sophisticated for a randomized controlled trial, and it increases the difficulty in implementing placebo acupuncture. The results of this trial cannot expel the placebo effect, so it may exaggerate the effect of acupuncture by using nontreatment as control. This trial will not assess the effect of acupuncture on the end points of live birth rate or clinical pregnancy rate for POR women due to limitations of short observation period and too many confounding factors during IVF-ET.

In conclusion, the results of this trial will show the effect of acupuncture versus nontreatment in increasing number of retrieved oocytes and improving ovarian reserve for women with POR. This study will contribute to the research of acupuncture for POR worldwide by publishing results in a peer-reviewed journal.

### Trial status

This trial is currently recruiting participants. The protocol version number and date: V1.0, May 9, 2018. The recruitment began on October 1, 2018. The estimated completion date of recruitment is February 29, 2021. The estimated study completion date is October 30, 2021.

## Data Availability

Not applicable.

## Abbreviations

POR: Poor ovarian response
COH: Controlled ovarian hyperstimulation
IVF: In vitro fertilization
ART: Assisted reproductive technology
GnRH: Gonadotrophin-releasing hormone
IVF-ET: In vitro fertilization-embryo transfer
DOR: Diminished ovarian reserve
POI: Premature ovarian insufficiency
FSH: Follicle-stimulating hormone
LH: Luteinizing hormone
AMH: Anti-Müllerian hormone
E2: Estradiol
AFC: Antral follicle count
TEAS: Transcutaneous electrical acupoint stimulation
RCT: Randomized controlled trial
SAS: Self-rating anxiety scale
AEs: Adverse events
